# A Giant Left Intraventricular Thrombus Associated With Apical Hypertrophic Cardiomyopathy Mimics Cancer

**DOI:** 10.7759/cureus.15554

**Published:** 2021-06-09

**Authors:** Nadia Raza, Shane Burnette, Fowrooz S Joolhar, Aslan Ghandforoush, Theingi Tiffany Win

**Affiliations:** 1 Internal Medicine, University of California, Los Angeles (UCLA) Kern Medical Center, Bakersfield, USA; 2 Internal Medicine/Cardiology, Kern Medical Center, Bakersfield, USA; 3 Cardiology, University of California, Los Angeles (UCLA) Kern Medical Center, Bakersfield, USA

**Keywords:** lv thrombus, left ventricle (lv) thrombus, apical hypertrophy cardiomyopathy, hypertrophy cardiomyopathy, giant lv thrombus

## Abstract

Hypertrophic cardiomyopathy (HCM) is a common disease that can be acquired due to chronic hypertension or via autosomal dominant inheritance. Several patterns of HCM have been described, of which a rare variant is apical hypertrophic cardiomyopathy (AHCM). Atrial thrombus is a well-recognized complication of HCM especially in the setting of atrial fibrillation (AF). However, left ventricular thrombus (LVT) formation is not nearly as prevalent as atrial thrombus. Here is a case of a 57-year-old Hispanic female with AHCM who presented with significant unintentional weight loss and unexplained anemia and was subsequently found to have a large left intraventricular mass suspicious for a tumor vs. ventricular thrombus. The diagnosis was complicated due to the large size of the mass and presenting symptoms suspicious of malignancy.

## Introduction

The development of a left ventricular thrombus (LVT) is a well-known complication in various cardiac conditions with the highest rate observed in acute anterior myocardial infarction and severe left ventricular (LV) systolic dysfunction. In a study of 949 eligible patients, the prevalence of LVT seen by transthoracic echocardiography (TTE) was 8.85% with the highest prevalence of 39.29% (33/84) observed in patients with dilated cardiomyopathy, followed by myocardial infarction with a prevalence of 29.76% (25/84) [1]. AHCM is a well-recognized cause of left atrial thrombus formation due to complications of atrial fibrillation (AF). However, LVT in HCM is extremely rare especially when the patient is in sinus rhythm [2], as seen in this case.

Malignancy is also an important consideration in the differential diagnosis of LV masses, especially when presenting with symptoms including fevers, night sweats, cachexia, and weight loss. Primary cardiac tumors are rare compared to secondary tumors. In general, cardiac metastases are considered to be rare; however, when sought for, the incidence seems to be not as low as expected, ranging from 2.3% to 18.3% [3]. Here we present a case of a 57-year-old Hispanic female with AHCM who presented with significant weight loss that was subsequently found to have a large left intraventricular mass suspicious for a tumor vs. ventricular thrombus. In this case report, we explore the relationship between apical hypertrophic cardiomyopathy (AHCM) and LV thrombus formation as well as work up for distinguishing the diagnosis of LV mass suspicious for cancer.

## Case presentation

The patient is a 57-year-old Hispanic female with hypertension, type 2 diabetes mellitus, hyperlipidemia, AHCM, and grade 3 diastolic dysfunction with preserved ejection fraction at 60% who presented to the emergency department (ED) complaining of bilateral lower extremity edema and lightheadedness since a day. Her history was significant for 76-pound weight loss over the past year. Her vitals were normal on presentation. 

**Figure 1:**
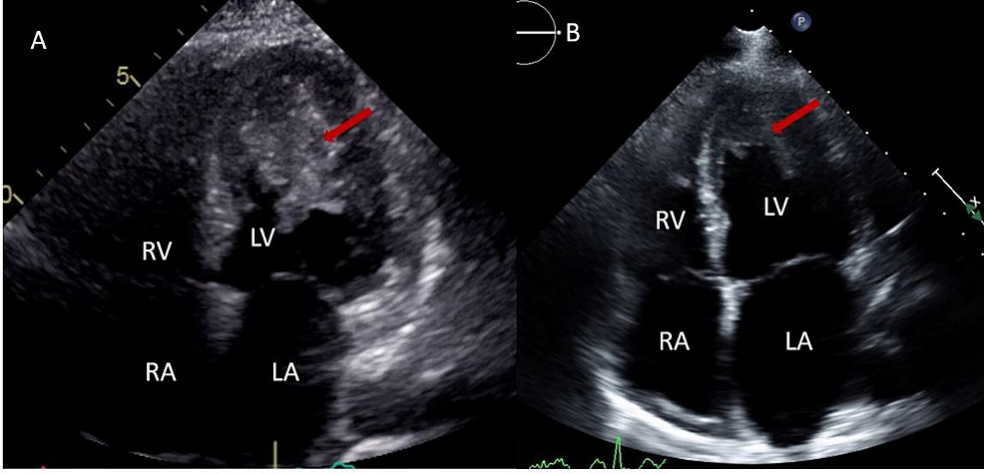
A TTE revealing a gigantic LV apical mass prior to anti-coagulation (left). A repeat TTE revealing significant resolution of LV thrombus after three months of anti-coagulation (right). TTE, transthoracic echocardiogram; LV, left ventricle; RV, right ventricle; LA, left atrium; RA, right atrium

**Figure 2:**
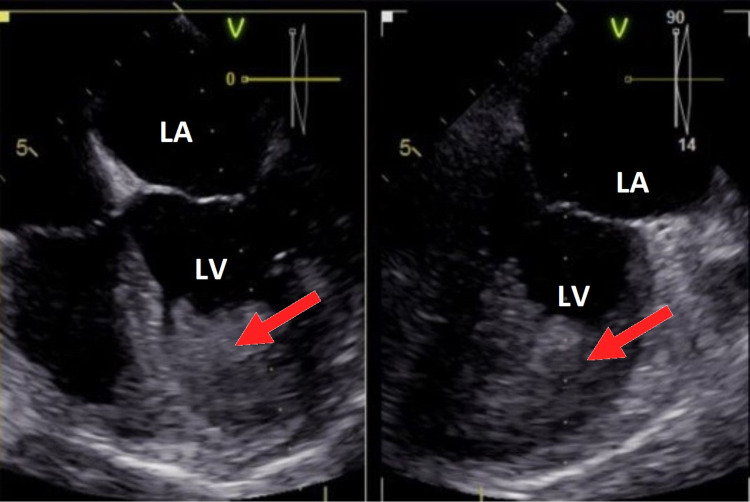
A TEE revealing a gigantic LV mass. TEE, transesophageal echocardiogram; LV, left ventricle; LA, left atrium

Her significant laboratory findings were hemoglobin 9.8 g/dL, brain natriuretic peptide 1119 pg/mL, albumin 2.9 g/dL, blood urea nitrogen (BUN) 26 mg/dL, and creatinine 1.14 mg/dL. The patient was in acute heart failure exacerbation and was started on IV diuretics. A TTE was ordered to evaluate the degree of heart failure, which revealed an incidental large partially mobile echo density extending from the mid-lateral wall to the apex and covering approximately one-third of the LV cavity measuring 4.8 cm x 3.4 cm as shown in Figures 1-2 and Video 1.

**Video 1:** A TTE revealing a gigantic LV apical mass. TTE, transthoracic echocardiogram; LV, left ventricle

**Figure 3:**
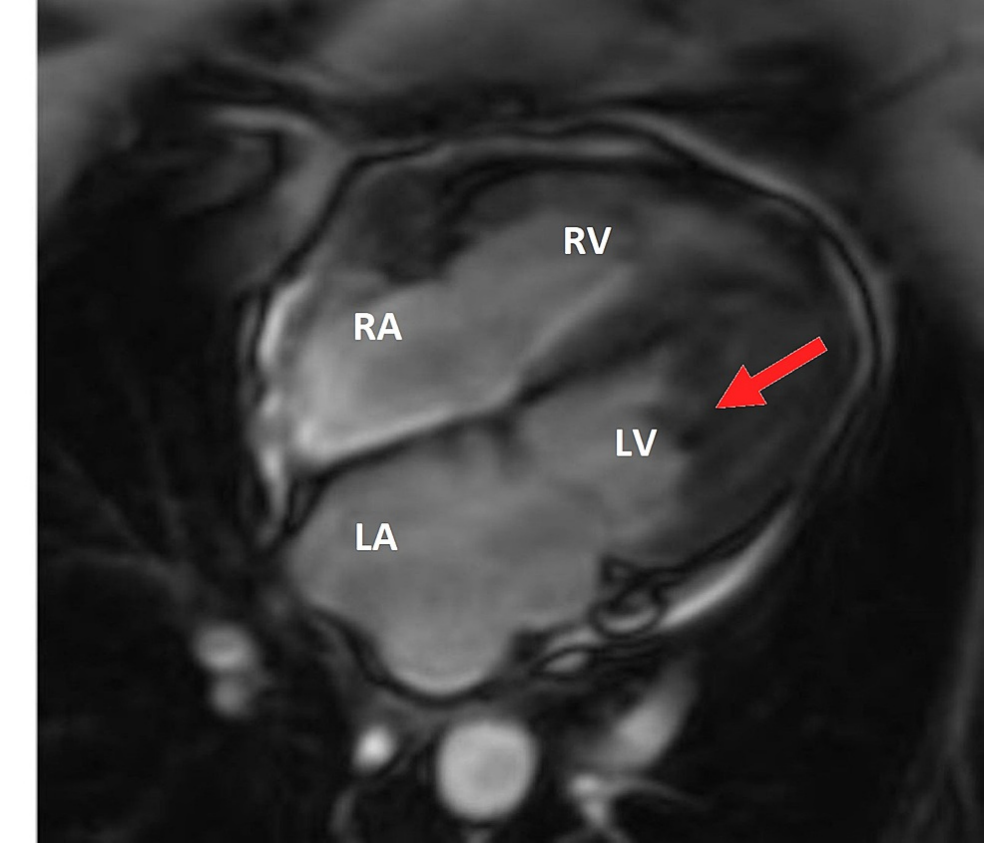
A cardiac MRI revealing a gigantic LV mass. LV, left ventricle; LA, left atrium; RV, right ventricle; RA, right atrium

The patient underwent further diagnostic imaging with a transesophageal echocardiogram (TEE) and CT of the chest, abdomen, and pelvis. TEE revealed a very large partially mobile mass in the LV that was either a tumor/mass or a thrombus as shown in Video 1. CT abdomen demonstrated large right hepatic heterogeneous mass, suspected hemangioma, and 2.5 cm right side adrenal mass. Further evaluation of LV mass with cardiac CT angiography showed central calcification of the mass. Cardiac MRI revealed a large LV mass from mid cavity to apex demonstrating late gadolinium enhancement involving the subendocardial LV myocardium from the mid to apical anterior and inferolateral subendocardial as shown in Figure 3. These findings were concerning for endomyocardial fibrosis or underlying neoplasm with superficial thrombus. Positron emission tomography (PET) CT performed failed to appreciate fluorodeoxyglucose (FDG) uptake of the known LV mass, thereby excluding high-grade malignancy but not excluding the possibility of a benign or low-grade tumor. The patient was placed on heparin throughout the hospital stay for possible thrombus, then bridged with warfarin with target INR at two to three. She was referred to medical oncology and after a multidisciplinary discussion, the decision was made to medically treat with warfarin and repeat TTE. A repeat TTE after a total of three-month therapy with warfarin resulted in nearly complete resolution of giant LV mass with no complication as shown in Figure 1 (right).

## Discussion

Hypertrophic cardiomyopathy has many known morphological variants including asymmetrical or diffuse that may be obstructing or non-obstructing. Additionally, a rare variant known as AHCM is found in about 3%-11% of the North American population [4]. AHCM may present with apical outpouching or aneurysm formation that may lead to an increased risk of thrombosis formation and subsequent thromboembolic events. The apical regions found in AHCM may develop ischemia and hypokinesis due to the increased oxygen demand of thick apical walls. This may lead to ischemia and dilatation with subsequent aneurysm formation. The hypokinetic areas may serve as a nidus for thrombus formation and may go unnoticed until late thromboembolic complications occur due to the emergence of resulting scar tissue. Moreover, when evaluating an LV mass, thrombus formation is highly suspected as this is the most common etiology, however, one should also include tumors, vegetations, and metastasis in the differential diagnosis [4]. Primary cardiac tumors are rare and are most commonly myxomas. The majority of myxomas are in the left atrium (LA) (75%) followed by the right atrium (RA) (20%), and LV (2.5%) [5]. Compared to primary tumors, metastasis to the heart is slightly more common in the general population with a frequency of tumors to the pericardium, myocardium, great vessels, or coronary arteries between 0.7% and 3.5%. Any tumor type is capable of metastasizing to the heart but the most common ones are lung ( 36%-39%), breast (10%-12%), and hematologic malignancies (10%-21%) [6].

Left ventricular thrombus is a potentially fatal complication that is implicated in LV dysfunction and seen after acute myocardial infarction, however, with advancements in percutaneous coronary interventions and medical goal-directed therapies, the incidence of LVT has significantly decreased. It has now become apparent that LVT may be implicated in other disease processes, such as heart failure, HCM, and AHCM. When evaluating an LV mass, transesophageal echocardiography has been broadly accepted as the primary screening modality. Other imaging modalities such as cardiac CT and cardiac MRI can provide additional information when echocardiography cannot delineate the extent of cardiac wall involvement [6].

In one study of patients identified by echocardiograms as having LVT, 68.5% had heart failure as a precipitating factor with 38% being de novo heart failure diagnosis with inadequate guideline-directed medical therapy with 86% having EF less than or equal to 40%, 8% having EF between 41% and 49%, and 5% having EF greater than or equal to 50% [7]. The second most common precipitating event was myocardial infarction (MI) (25.9%) with 71% of those being ST-segment elevation MI implicating the left anterior descending artery (LAD) in 77.8% of cases, followed by a left main, right coronary, and left circumflex, each at 7.5% of cases [7]. From these studies, it appears that LVT with normal LV systolic function is rare which may explain why routine anticoagulation in heart failure has not proven beneficial. Furthermore, for these patients, they usually present with late complications such as embolic disease and have identifiable medical conditions that carry an increased risk for thromboembolic diseases such as connective tissue disease, ulcerative colitis, rheumatoid arthritis, dermatosis, or malignancy.

Although potential causes of LVT are well understood, namely pathologies that reduce ventricular contractility, cause local myocyte injury and hypercoagulable states, the pharmacological interventions are far less understood. In the GISSI-3 trial, patients who underwent treatment for MI with beta-blockers did not show a significant reduction in the incidence of LVT formation as compared with those who did not receive treatment [8]. Current recommendations for post-MI patients are the use of aspirin and a P2Y12 inhibitor. It would be rational to assume that this may reduce the occurrence of LVT thrombus; however, sufficient studies have not been done, therefore, in order to reduce the occurrence of LVT post-MI prompt revascularization is crucial.

Regardless of etiology, current treatment strategies for LVT include oral anticoagulation (direct oral anti-coagulants, DOAC) or warfarin. Current recommendations from the American College of Cardiology Foundation/American Heart Association (ACCF/AHA) state that warfarin is a reasonable therapy of choice for patients with ST-elevation MI as well as asymptomatic LVT, however, as per the American College of Cardiology/American Heart Association (ACC/AHA) guidelines there is no definite duration for treatment [9]. Whereas ACCP guidelines recommend warfarin plus low-dose aspirin (75-100 mg daily) for the first three months followed by discontinuation of warfarin and continuation of anti-platelet therapy, over single anti-platelet therapy or dual anti-platelet therapy (DAPT) for patients not requiring stent. Whereas for patients requiring bare-metal stent placement, triple anti-platelet therapy for one month is recommended over DAPT; warfarin and single anti-platelet therapy for the second and third month with discontinuation of warfarin thereafter and resumption of DAPT for up to 12 months. Furthermore, for patients requiring drug-eluting stents, triple therapy for three to six months with discontinuation of warfarin thereafter and continuation of DAPT for up to 12 months is recommended [10].

Although the current treatment for LVT is not completely agreed upon, anticoagulation for three to six months after identification of an LVT is reasonable. After the treatment period is finished, repeat imaging should be considered to determine if the thrombus had resolved. If no thrombus is identified anticoagulation should be stopped. If a new thrombus appears on echocardiography, continued anticoagulation should be administered. The difficult decision lies regarding whether an LV mass is still identified on repeat imaging. Differentiating between residual thrombus and a new thrombus may be difficult to determine from echocardiography findings, therefore, it would be reasonable to assess the mass with cardiac MRI (CMRI) as that would help differentiate acute vs. old thrombus. In that acute thrombus would show high signal intensity on T1 and T2 weighted imaging while older thrombus shows low signal and may show evidence of calcification [11]. From this point, clinicians should balance between the risks and benefits with regard to the continuation of anticoagulation when a residual old thrombus is identified. The role of non-vitamin K oral anticoagulants in the treatment of LVT is limited to case reports and case series, therefore, warfarin continues to be the mainstay of treatment [12-18].

## Conclusions

Identification of LV mass requires a broad differential from thrombus formation to malignant and benign cancers. Understanding risk factors, precipitating events and imaging findings help aid in differentiating the diagnosis. Once an LVT is identified, guidelines recommend anticoagulation using warfarin, with a reasonable goal of three to six months. Further anticoagulation should be based on clinical presentation while balancing the risks and benefits. We present a case of a de novo LVT identified on imaging that was completely resolved after three months of anticoagulation with warfarin without thromboembolic complications.
